# Comparative cost-effectiveness of immunoassays and FLOTAC for diagnosing *Giardia* spp. infection in dogs

**DOI:** 10.1186/s13071-019-3425-8

**Published:** 2019-04-08

**Authors:** Paola Pepe, Davide Ianniello, Leucio Camara Alves, Maria Elena Morgoglione, Maria Paola Maurelli, Antonio Bosco, Giuseppe Cringoli, Laura Rinaldi

**Affiliations:** 1 Department of Veterinary Medicine and Animal Production, University of Naples Federico II, CREMOPAR, Campania Region, Eboli, Italy; 2 Department of Veterinary Medicine, Federal Rural University of Pernambuco, R. Manuel de Medeiros s/n, Dois Irmãos, Recife, PE 52171-900 Brazil

**Keywords:** *Giardia* spp., Immunoassays, Diagnosis, FLOTAC

## Abstract

**Background:**

*Giardia* spp. is a protozoan pathogen and is the most common enteric parasite of domestic animals and humans. Assays for detecting infection in fecal samples using direct or indirect examinations are important tools for diagnosing the disease. The objective of the present study was to compare the cost-effectiveness of immunoassays and FLOTAC technique for diagnosing *Giardia* spp. infection in dogs.

**Results:**

Fecal samples from 80 positive stray dogs were tested for the presence of copro-antigens of *Giardia* spp. using the direct immunofluorescence assay (IFA), a rapid enzyme-linked immunosorbent assay (ELISA) and the FLOTAC double technique. All methods were performed in accordance with the instructions reported in the original description for each technique. The results showed that ELISA can be run in less time than IFA and almost at the same time of the FLOTAC technique. Among the tests used in this study, FLOTAC had the lowest cost per correct diagnosis, compared with immunoassays.

**Conclusions:**

The results from this cost-effectiveness analysis, in combination with the sensitivity and specificity of the FLOTAC technique, suggest that the FLOTAC technique can be use in the routine diagnosis of *Giardia* spp. infection in dogs.

## Background

Among protozoal infections, giardiasis is the most common disease in a wide variety of animals, including humans [1]. Once a person or animal has become infected with *Giardia* spp., zoonotic transmission cycles may occur [2]. In fact, new evidence has shown that there is a strict genetic relationship between some *G. duodenalis* genotypes isolated from infected humans and dogs [3]. In particular, assemblages A (subtypes I and II) and B (subtypes I and IV) have been associated with human infections [4], but are also found in a number of other mammalian hosts [5], and assemblage C from dogs was found in humans in Europe [6], thus suggesting the possibility of interspecies transmission [7].

*Giardia* infection in dogs is an important disease in veterinary medicine [8] and infected animals show clinical signs of disease two to three weeks after infection [9], characterized by diarrhea, vomiting, weight loss and lethargy. However, some animals do not present any clinical signs [10]. Surveys on a variety of canine populations have revealed a prevalence of *Giardia* infection ranging from 10% among well-cared-for dogs to 36–50% among puppies and up to 100% among kennel dogs, which are at highest risk of disease transmission [11]. Many factors appear to affect the prevalence of the infection including the animal’s characteristics (age, living conditions, animal density, nutritional status and immune status) and the diagnostic test used [12].

Assays for detecting infection in fecal samples using direct or indirect examinations are important tools for diagnosing the disease [13–15]. In general, the diagnosis is based on the detection of *Giardia* cysts (and occasionally trophozoites) in the feces of infected dogs [16]. The traditional approaches, such as use of fecal smears and flotation in tubes, have significant limitations due to the small size of the cysts. Moreover, shedding of cysts is intermittent, even in chronically infected individuals, thus requiring multi-day fecal examination [17]. Therefore, more sensitive diagnostic immunoassays such as the immunofluorescence assay (IFA; regarded as the “gold standard”), immunochromatography and the enzyme-linked immunosorbent assay (ELISA) [18] have been recognized as important tools for detecting *Giardia* spp. in fecal samples from dogs.

A new technique known as FLOTAC has been developed and proposed for diagnosing enteric parasites in animals and humans. In several studies, it has been shown to have high sensitivity, specificity and accuracy [19]. The aim of this study was to compare the cost-effectiveness of immunoassays and FLOTAC technique for diagnosing *Giardia* spp. infection in dogs.

## Results

Among the 80 samples examined, all (100%) were found to be positive by the FLOTAC test. The only test that revealed a negative sample was ELISA.

The costs of all the kits were ascertained based on an internet survey of the commercial kits available for diagnosing *Giardia* spp. (Table 1). The time taken to analyze the samples using each of the techniques and the sensitivity and specificity of each diagnostic test kit for *Giardia* spp. are shown in Tables 2 and 3, respectively.

**Table 1:** Direct costs of diagnosis kits of *Giardia* infection based on an internet survey of commercial kits

Test	Mean (US$)	Minimum (US$)	Maximum (US$)
ELISA	11.4	8.71	16.3
IFA	9.8	7.20	14.6
FLOTAC	1	0.50	1.5

**Table 2:** *Giardia* infection according to target of test, time and cost by sample

Test	Target	Result (min)	Cost/sample
ELISA	Antigen	11–12	+++
IFA	Antigen	40–50	++
FLOTAC	Parasite	12–15	+

*Note*: One sample is required for all tests

**Table 3:** Evaluation of immunoassay tests and FLOTAC technique compared to the immunofluorescence antibody test as a gold test in diagnosis of *Giardia* spp. infection in dogs

Parameter (%)/technique	ELISA	FLOTAC
Sensitivity	98.75	100
Specificity	100	100
True prevalence	100	100
Estimated prevalence	98.00	100
Predictive value (+)	100	100
Predictive value (−)	0	0
Accuracy	98.75	100
Incorrect classification	1.25	0

Comparing the sensitivity and specificity of these tests, FLOTAC and IFA have the same capability to diagnose *Giardia* spp. infection in dogs but the FLOTAC technique showed higher sensitivity than ELISA. The Kappa test showed a good and a very good agreement of 1.00 (IFA/FLOTAC) and 0.98 (IFA/ELISA), respectively.

## Discussion

The coproparasitological diagnostic tests for *Giardia* spp. in dogs that have been used include direct smears, fecal flotation, centrifugal fecal flotation, IFA, ELISA and polymerase chain reaction (PCR) assay. These tests can be used either alone or in combinations in order to improve the sensitivity [20]. Moreover, it has been reported that to make a true diagnosis of *Giardia* spp. infection in dogs, immunoassays need to be used because the sensitivity and specificity of these tests are higher [17]. One-step ELISA and immunofluorescence assays have been recognized as important tools for detecting *Giardia* spp. in fecal samples from dogs [17, 21–23].

Several studies comparing diagnostic tests for *Giardia* spp. infection in dogs have shown that parasitological tests and immunoassays have similar performance [24] and that they need to be used together [25]. On the other hand, almost all studies have shown that immunoassays were more sensitive and that they improved the accuracy of diagnosing *Giardia* spp. infection in dogs [17, 21–23, 26–28].

IFA is the serological test that is most used for diagnosing *Giardia* spp. infection, given that it is regarded as the gold-standard test. ELISA is also widely used, not only because it is a highly sensitive and specific test, but also because it is very easy to use. Since immunoassays detect antigens, it can be expected that both ELISA and IFA would detect more dogs as positive than would tests based on cysts, such as the FLOTAC technique.

However, in the present study, it was observed that the FLOTAC technique showed the same sensitivity and specificity as IFA and a higher sensitivity than ELISA.

Dog owners generally associate giardiasis when their pets presenting the symptom of diarrhea. However, some animals remain asymptomatic [16] and sometimes they are erroneously treated. These animals present lower numbers of cysts in stool samples and false-negative test results may occur. In this regard, antigen tests are not indicated for the follow-up of patients with persistent symptoms after being treated for giardiasis, because the test sensitivity is compromised [29].

For detection of intestinal protozoa, the FLOTAC technique has been reported as a promising test in comparison with other parasitological techniques [30]. Furthermore, Speich et al. [31] made a comparative cost assessment of the FLOTAC and Kato-Katz techniques for diagnosing soil-transmitted helminths, and they found that the cost of the FLOTAC technique was higher than the cost of Kato-Katz, when salaries and costs due to materials and infrastructure were included. However, to our knowledge, the present study is the first to compare FLOTAC with immunoassays.

To make a diagnosis of dog’s giardiasis, the laboratories should have the necessary equipment for the accomplishment of the tests, particularly for immunoassays. All three techniques, IFA and ELISA and FLOTAC, can be performed at an unsophisticated laboratory, but FLOTAC is a diagnostic tool that is easy to apply for routine diagnosis of *Giardia* spp. infection in dogs.

Analysis on the time taken and the samples required for making the diagnosis using each of the techniques showed that ELISA could be run in a shorter time than IFA and that this time was closer to that required for the FLOTAC technique (Table 2). The time that has elapsed between the onset of clinical signs and making the diagnosis of giardiasis in dogs is an important point because some animals show severe diarrhea that may be fatal if left untreated.

Among the tests used in this study, FLOTAC had the lowest cost per correct diagnosis, in comparison with the immunoassays (Table 1).

In this study there was very good agreement between results obtained with IFA and FLOTAC. The discordance between IFA and ELISA assay can be explained by the detection limits for this test, which detects cyst wall proteins [32].

## Conclusions

The results from this cost-effectiveness analysis, in combination with the sensitivity and specificity of the FLOTAC technique, suggest that the FLOTAC technique can be used in making routine diagnoses of *Giardia* spp. infection in dogs.

## Methods

The objective of the present study was to compare the cost-effectiveness of immunoassays and the FLOTAC technique for diagnosing *Giardia* spp. infection in dogs. A total of 80 positive fecal samples according to the gold-standard IFA test, were included in this study. All samples were from stray dogs living in the city of Naples (Campania region, southern Italy) that had been brought to the veterinary Hospital of the School of Veterinary Medicine. The technicians were blinded to patient history and results of tests.

Three methods were used: the IFA test using a MeriFluor^®^ *Cryptosporidium/Giardia*, (Meridian Bioscience Diagnostic, Cincinnati, OH, USA), a rapid ELISA using the IDEXX SNAP^®^ test (Idexx Laboratories Inc., Schiphol-Rijk, Netherlands), and the FLOTAC double technique [19] in which zinc sulfate (specific gravity = 1.350) was used as the flotation solution. Magnifications of 100× and 400× were used to identify protozoan cysts. The results were expressed as the arithmetic mean of the number of cysts per gram (CPG) of feces. In order to evaluate cost-effectiveness, the IFA test was used as the gold-standard test.

In using IFA, the numbers of *Giardia* spp. cysts found were ranked into the following three levels: 1 (1 cyst); 2 (1–2 cysts); and 3 (3–4 cysts) per reading area.

All methods were performed in accordance with the manufacturer’s instructions. The sensitivity, specificity, positive predictive value (+PV), negative predictive value (-PV), accuracy, true estimated prevalence and incorrect classification were determined in comparison to the IFA technique as the gold standard. The InStat software 3.01 (GraphPad Software, Inc., San Diego, California, USA) was used to calculate all parameters. To assess the cost-effectiveness of the immunoassays and the FLOTAC it was considered that laboratories would have all the necessary equipment to undertake the tests. To calculate a measure of agreement between IFA, ELISA and FLOTAC, the results were assessed using Cohn’s Kappa coefficient with 95% confidence interval.

## Abbreviations

IFA: immunofluorescence antibody test
ELISA: Enzyme-linked immunosorbent assay
CPG: cysts per gram
